# End-to-end prediction of clinical outcomes in head and neck squamous cell carcinoma with foundation model-based multiple instance learning

**DOI:** 10.1186/s44398-025-00003-8

**Published:** 2025-06-24

**Authors:** Asier Rabasco Meneghetti, Marta Ligero Hernández, Jens-Peter Kühn, Steffen Löck, Zunamys Itzell Carrero, Raquel Perez-Lopez, Keno K. Bressem, Titus J. Brinker, Alexander T. Pearson, Daniel Truhn, Sven Nebelung, Jakob Nikolas Kather

**Affiliations:** 1 Else Kroener Fresenius Center for Digital Health, Faculty of Medicine and University Hospital Carl Gustav Carus, TUD Dresden University of Technology, Dresden, 01307 Germany; 2 German Cancer Consortium (DKTK), Partner Site Dresden, German Cancer Research Center (DKFZ), Heidelberg, Germany; 3 Institute and Policlinic for Diagnostic and Interventional Radiology, Faculty of Medicine and University Hospital Carl Gustav Carus, Technische Universität Dresden, Dresden, Germany; 4 OncoRay – National Center for Radiation Research in Oncology, Faculty of Medicine and University Hospital Carl Gustav Carus, Technische Universität Dresden, Helmholtz-Zentrum Dresden-Rossendorf, Dresden, Germany; 5 Department of Radiotherapy and Radiation Oncology, Faculty of Medicine, University Hospital Carl Gustav Carus, Technische Universität Dresden, Dresden, Germany; 6 National Center for Tumor Diseases (NCT), Partner Site Dresden, Germany: German Cancer Research Center (DKFZ), Heidelberg, Germany; 7 Faculty of Medicine and University Hospital Carl Gustav Carus, Technische Universitat Dresden; Helmholtz-Zentrum Dresden-Rossendorf, Dresden, Germany; 8 Radiomics Group, Vall d’Hebron Institute of Oncology, Vall d’Hebron Barcelona Hospital Campus, Barcelona, Spain; 9 Department of Diagnostic and Interventional Radiology, Technical University of Munich, School of Medicine and Health, Klinikum Rechts Der Isar, TUM University Hospital, Ismaninger Str. 22, Munich, 81675 Germany; 10 Department of Cardiovascular Radiology and Nuclear Medicine, Technical University of Munich, School of Medicine and Health, German Heart Center, TUM University Hospital, Lazarethstr. 36, Munich, 80636 Germany; 11 Digital Biomarkers for Oncology Group, German Cancer Research Center (DKFZ), INF 223, Heidelberg, 69120 Germany; 12 Section of Hematology/Oncology, Department of Medicine, University of Chicago, Chicago, IL USA; 13 Department of Diagnostic and Interventional Radiology, Medical Faculty, RWTH Aachen University, Aachen, 52074 Germany; 14 Department of Medicine I, University Hospital Dresden, Dresden, Germany; 15 Medical Oncology, National Center for Tumor Diseases (NCT), University Hospital Heidelberg, Heidelberg, Germany

**Keywords:** Foundation models, Head and neck cancer, Prognosis, Radiomics, Multimodality

## Abstract

**Background:**

Foundation models have shown promise in medical AI by learning flexible features from large datasets, offering new opportunities for improving endpoint prediction. However, usage of foundation models for endpoint prediction using routine imaging in head and neck squamous cell carcinoma patients remains unexplored. Within this study, we evaluated the potential of foundation-model based multiple instance learning for prediction of 2-year overall survival, locoregional control and freedom from distant metastasis across three external head and neck squamous cell carcinoma patient cohorts using 2D, multiview and 3D approaches while comparing prediction and stratification performance with handcrafted radiomics and clinical baselines.

**Results:**

2D multiple-instance learning models achieved 2-year test area under the receiver-operator curve (AUROC) range of 0.75–0.84 for 2-year overall survival, 0.66–0.75 for 2-year locoregional control and 0.71–0.78 for 2-year freedom from distant metastasis across three different external cohorts, outperforming multiview and 3D multiple instance learning models (AUROC range: 0.50–0.77, p $\ge$ 0.15) and showing comparable or superior performance to handcrafted radiomics (AUROC range: 0.64–0.74, p $\ge$ 0.012). Significant stratification was observed from the 2D MIL models (hazard ratios: 2.14–4.77, p $\le$ 0.039). 2D MIL models were also shown to learn endpoint-specific correlation patterns such as N-stage for 2-year freedom from distant metastasis prognosis. Multimodal enhancement of 2-year OS/FFDM (AUROC range: 0.82–0.87, p $\le$ 0.018) for patients without human papilloma virus positive tumors.

**Conclusions:**

FM-based 2D MIL demonstrates promise in HNSCC risk prediction as well as stratification of clinical outcomes. The models match or outperform radiomics baselines, learning clinically-related patterns and showing enhancement of clinical baselines in non-human papilloma virus positive patients.

**Supplementary Information:**

The online version contains supplementary material available at 10.1186/s44398-025-00003-8.

## Background

Despite multimodal treatment, overall survival (OS) at 5-years remains at 30–70% for patients with head and neck squamous cell carcinoma (HNSCC), with existing prognostic methods based on the TNM staging system [1]. While human papilloma virus (HPV) infection status is the only validated biochemical test for HNSCC, with HPV16 + oropharyngeal tumors being more radiosensitive and presenting better response to treatment [2], there is currently no consensus for its integration into treatment planning. Factors such as extranodal extension have been shown to have a negative prognostic effect but are not consistently quantified in a unified way [3]. This highlights the need for more precise prognostic biomarkers that can stratify patients into risk groups and support patient-tailored treatments [4] such as dose de-escalation treatment for radiotherapy to reduce patient toxicity in low-risk patients [5] or detection of persistent tumors to consider for second-line treatment options [6].

CT scans are routinely acquired for every patient with HNSCC and have been proposed as sources for extracting quantitative, non-invasive biomarkers. Radiomics was initially devised to extract handcrafted features from radiological images to create such image-based biomarkers [7]. Despite numerous studies and standardization efforts [8], the adoption of such handcrafted radiomics biomarkers in clinical settings remains limited. One of the main drawbacks of such studies is the time-intensive requirement for manual image annotations and the variability across studies attempting to predict the same outcome [9]. In contrast, deep learning (DL) approaches offer flexibility by learning image representations without the need for prior handcrafted feature definition. DL methods have been developed for clinical applications in HNSCC to predict locoregional control (LRC) from PET/CT images [10], epithelial growth factor receptor (EGFR) mutation status [11] or HPV infection in oropharyngeal cancer patients [12] with CT. However, DL methods typically require larger datasets for training models than handcrafted radiomics studies, which limits the implementation of such methods in practice where data size can be limited.

An emerging area of AI research in medicine is the development and use of foundation models (FMs), models trained on large and unlabeled datasets to capture general patterns in the data. FMs can serve as frozen backbones for downstream tasks, bypassing the need to train models from scratch. This approach facilitates the extraction of general-purpose features and the development of predictive DL models from smaller datasets. Currently available FMs for feature extraction that utilize radiological images for training include BioMedClip [13], which uses 2D images from single views or the FM by Pai et al. [14] which employs 3D bounding boxes. Some of the current FMs require aggregation methods to analyze CT scans. Multiple instance learning (MIL) is a weakly-supervised paradigm [15] that addresses this need by creating groups of data instances from each patient [16]. MIL allows for the simultaneous learning of relationships between different data instances, such as the different 2D slices of a CT image, and a target, such as the patient's outcome. This is potentially advantageous as it enables handling data with ambiguous labels or identification of relevant patterns when extensive labeling of specific regions or pixels is impractical. DL-based MIL has been successfully employed within the histopathology domain for tasks such as microsatellite instability classification in colorectal cancer [17] or immunotherapy response on solid tumors [18]. In the radiological domain, MIL has been explored [19, 20] but has not been employed before for outcome prediction using pretreatment CT images in HNSCC with FMs features.

The present study aims to implement, for the first time, DL-based MIL approaches applied to FM features from pretreatment CTs of HNSCC patients for outcome prediction. As we hypothesize that MIL can aggregate FM features to learn prognostic biomarkers from the CT images, we conduct a comprehensive study including different FMs as feature extractors combined with MIL approaches to predict three different endpoints for HNSCC. The results of this study are evaluated across three different test cohorts and compared with handcrafted radiomics models and a clinical baseline. Finally, we explore correlations with clinical factors as well as the enhancement our models provide to the clinical baseline for prognosis in the overall patient population as well as non-HPV + HNSCC patients.

## Methods

### Patient cohorts and endpoint dichotomization

This retrospective study considers three cohorts extracted from The Cancer Imaging Archive (TCIA) [21]: the RADCURE [22], HN-PET-CT [23, 24], and the HN1 cohorts [7, 25]. The RADCURE cohort comprises 3346 head and neck cancer patients treated with definitive radiotherapy or radiochemotherapy between 2005 and 2017 at the Princess Margaret Cancer Centre, Toronto, Canada. The HN-PET-CT cohort consists of 298 HNSCC patients from 4 different hospitals in Canada. These patients had received either definitive radiotherapy, radiochemotherapy or a combination of surgery and either adjuvant radiotherapy or radiochemotherapy between 2006 and 2014. The HN1 cohort includes 137 HNSCC patients treated with radiotherapy or radiotherapy with concurrent chemotherapy at the MAASTRO clinic in Maastricht, the Netherlands, starting from 2004. The study was conducted in accordance with the TRIPOD + AI statement.

**Fig. 1:**
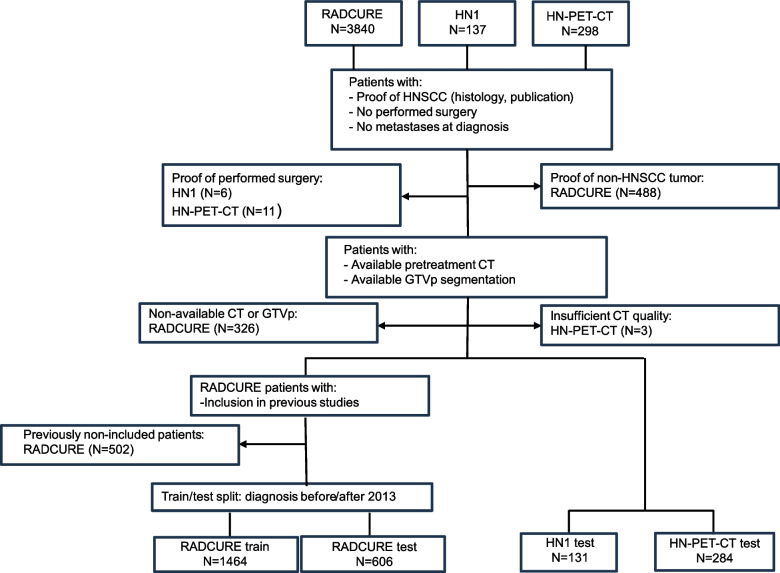
Flowchart showing inclusion and exclusion criteria for study cohorts as well as cohorts used for training models and for testing. Excluded patients are shown per cohort and the reason for the exclusion

Inclusion and exclusion criteria for the study are summarized in Fig. 1. Patients who underwent surgery, presented with metastases upon diagnosis, or that lacked either histological confirmation or documented diagnosis in their source publications for HNSCC were excluded. For endpoint dichotomization, patients who were lost to follow-up before the 2-year mark or had ambiguous endpoint data, such as presenting recurrence the same day of treatment, were excluded from the corresponding endpoint analysis. Patients with persisting disease were excluded from the 2-year locoregional control (LRC) endpoint. Endpoints were OS, LRC, and freedom from distant metastasis (FFDM), all dichotomized at the 2-year mark similarly to a prior study [26]. OS was defined as the time from treatment start to death by any cause or loss to follow-up. LRC was defined from treatment start to local or regional tumor recurrence or loss to follow-up. FFDM was defined from treatment start until detection of distant metastasis or loss to follow-up. Inclusion criteria were defined as the availability of pretreatment CTs and primary gross tumor volume (GTVp) segmentations.

Included patients were previously reported in studies [7, 24, 26] on outcome prediction for OS [7, 26] or LRC and FFDM [24], with either non-MIL DL or handcrafted-radiomics approaches. In contrast, the current study focuses on the application of FMs as feature extractors for MIL modeling of 2-year endpoint prediction, stratification, and multimodal integration alongside clinical variables for OS, LRC and FFDM in HNSCC patients.

### Study design

**Fig. 2:**
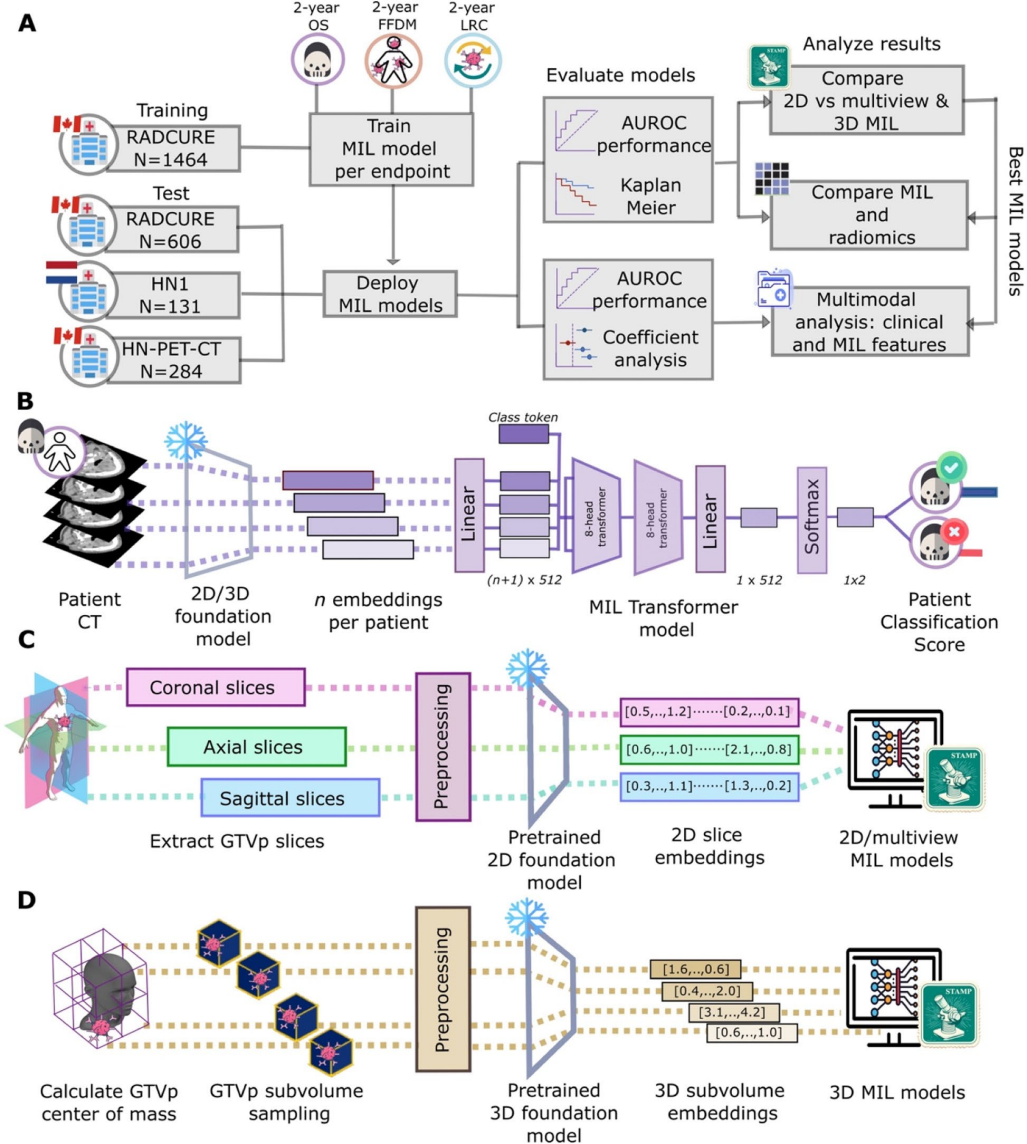
Workflow for overall study design, modeling and extraction. MIL models were trained to predict one of three 2-year endpoints on the training cohort and evaluated on three test cohorts (**A**) for each of three 2-year endpoints. For 2-year OS (**B**), for example, a patient would have embeddings extracted from the CT which would be used as input into a MIL transformer, learning simultaneously on embeddings from all CTs slices/subvolumes and pooling them to return patient-level scores for 2-year OS. Embeddings are extracted from FMs using either (**C**) axial or multiple views of CT slices containing the GTVp or from (**D**) subvolumes sampled around the GTVp center for a 3D approach

Within this retrospectively-designed study as shown in Fig. 2, cohorts were divided into training and test cohorts to train and test 2D, multiview and 3D MIL models with FM backbones for HNSCC outcome prediction and stratification, to test whether MIL models outperformed handcrafted radiomics features and whether MIL scores added value to already-available clinical baselines (Fig. 2A). In order to obtain features from the CT images to be used in MIL modeling (Fig. 2B), two approaches were considered for feature extraction: a 2D approach to extract information (Fig. 2C) from either the axial view or from the three different views of the CT image and a 3D approach based on sampling volumes within the CT around the GTVp (Fig. 2D).

### Preprocessing and feature extraction

For all CT images, the patient body was segmented through TotalSegmentator [27] and used to mask out the patient bed.

For 2D MIL, all 2D CT slices containing the GTVp mask were selected for each CT view. CT images and GTVp segmentations were extracted and resampled to a voxel spacing of 1 × 1x1 mm^3^ through the SimpleITK Python library. Hounsfield Units (HU) were clipped using a soft tissue windowing approach with a level of 50 HU and a window width of 120 HU. Images were converted to grayscale, cropped around the GTVp center of mass to a size of 224 × 224 pixels and normalized according to FM preprocessing parameters (Supplementary Table 1). Different pretrained feature extractors were considered to obtain slice-level representations of the patient: i) ImageNet-pretrained ResNet50, ii) ImageNet-pretrained SwinViT [28], and iii) the PMC-15 pretrained vision encoder extracted from the BioMedClip FM [13]. Models were constructed from either the axial view alone (2D models) or from all three CT views (multiview models).

For 3D MIL, for the FM by Pai et al. [14] images were preprocessed according to FM specifications of the original publication: HU values were normalized to a range of −1024 and 2048 HU and resampled to a voxel spacing of 1 × 1x1 mm^3^. To capture different contexts of the tumor and of its surrounding tissue, 50 × 50x50 voxel volumes were generated by randomly sampling 100 instances from a multivariate normal distribution with a variance of 16 and with the coordinates of the center of mass of the GTVp as the mean. An ImageNet-pretrained inflated 3D ResNet50 [29] was also employed, with features extracted similarly to the previously mentioned FM and using the same HU windowing.

### MIL model training

Extracted features were input into a MIL Transformer [30, 31] using the STAMP package version 1.0.1. A separate MIL model was trained for each endpoint using class-stratified fivefold cross-validation on the training cohort. For each MIL model and endpoint, the model whose AUROC was closest to the median AUROC across the cross-validation holdout set was chosen for deployment in the test cohorts. Subgroup analysis were also conducted to assess performance changes based on contrast enhancement status.

Training was performed for 64 epochs with a patience parameter of 15 for early stopping. Optimization followed default STAMP settings, using the ADAM optimizer with initial learning rate of 1e-3, a maximum allowed learning rate of 1e-4 and momentum of 0.95. Learning rate scheduling was performed through cosine annealing. Weights and folds for CV were initialized with a fixed seed of 1337. Model training and checkpointing were guided through the use of a weighted binary cross-entropy loss function with weights set to the inverse class proportion. Checkpointing was based on loss reduction within the holdout CV set.

For each extracted CT slice or subvolume, an augmented version was also created by applying a random rotation (0 to 10 degrees) and Gaussian blur applied with a probability of 0.5. Both augmented and non-augmented features were used for model training. At each epoch, either augmented or non-augmented features were passed into the model with a probability of 0.5. Only non-augmented features were employed for performance evaluation purposes such as checkpointing during training and model deployment in external cohorts. All models were built using PyTorch 2.2.0 and trained on an NVIDIA RTX A100 48 GB GPU.

### Handcrafted radiomics extraction and modeling

Morphological, statistical, and texture features were extracted alongside from the base image using PyRadiomics 1.3.0 with only IBSI-compliant features included. Feature selection consisted of variance filtering, z-score standardization, hierarchical clustering with correlation distance cutoff of 0.20 to define cluster membership, average Spearman correlation for cluster representative selection and minimum redundancy maximum relevance selection similarly to a prior study for HNSCC handcrafted radiomics modeling [32]. The number of features selected was set to the number of samples in the minority class divided by 10. Selected features were input into a logistic regression model for training and evaluated on the test cohorts. Subgroup analysis were again conducted to assess performance changes based on contrast enhancement status. Extraction parameters are shown in Supplementary Table 2.

### Clinical baseline and multimodal enhancement

Baseline clinical models were developed using known prognostic variables for HNSCC: T stage, N stage and HPV status as well as clinicodemographic factors including age, gender and treatment regime. Models were trained using a logistic regression with balanced class weights implemented through scikit-learn default parameters. Relationships between MIL scores and clinical baseline features were assessed by the coefficients of a multivariate logistic regression model containing the MIL scores and the clinical baseline features on the training cohort as well as with a correlation analysis between MIL scores and clinical baseline variables in the external test cohorts. The enhancement in model predictive performance through inclusion of MIL scores was assessed in all patients of the test cohorts as well as in the subgroup of patients without HPV + tumors through a logistic regression with balanced class weights.

### Statistical analysis

Statistical comparisons between categorical variables in the training and test cohorts were conducted using the chi-squared test or Fisher’s exact test. Continuous variables were compared using the Mann–Whitney *U*-test. The AUROC was chosen as a summary metric for evaluating classification performance on 2-year endpoints. AUROC 95% confidence intervals (95% CI) were estimated from the empirical distribution through 1000 class-stratified bootstraps. Risk group stratification was performed using the Kaplan–Meier (KM) estimator, with the 2-year MIL scores as surrogate time-to-event risk scores. The cutoff was defined as the median score from the training cohort, and risk group separation was assessed through the Cox hazard ratio (HR) and the logrank test *p*-value. For MIL model explainability, attention scores were generated and the top three CT slices from selected high-risk patients were displayed using an element-wise gradcam to highlight the image regions important for modelprediction within the slice through the gradcam Python library.

AUROC differences were tested through score permutation tests: for 1000 bootstraps, predictions from randomly selected patients were permuted between models to define a null distribution and heuristic *p*-value. All statistical tests were conducted in a two-sided manner unless specified. Significance of MIL scores in multivariate logistic regression was evaluated through significance of their log-odds ratios (LOR). Imputation of missing clinical factors was performed based on mode or mean for categorical or numerical variables respectively. Correlations between clinical baseline factors and MIL scores were assessed using Spearman rho, or the point-biserial correlation for numerical-binary variable pairs. A *p*-value threshold of 0.05 was chosen for significance. Multiple comparison adjustment was performed through the Benjamini–Hochberg method. All analyses were performed in Python 3.11 or R version 4.4.0.

### Code availability

The code containing the models and feature extraction pipeline are available at: https://github.com/KatherLab/HNSCC_FM_MIL_study. The STAMP code used for the analysis within this paper can be found at https://github.com/KatherLab/STAMP

## Results

### Patient cohort clinical characteristics

The characteristics of the patients in the RADCURE train (*N* = 1404), RADCURE test (*N* = 606), HN1 (*N* = 131), and HN-PET-CT (*N* = 284) cohorts are shown in Table 1. The RADCURE test cohort patients had significantly lower ECOG stages, higher N-stages, and a higher proportion of oropharyngeal tumors (all *p* < 0.001). The HN1 cohort had a significantly higher proportion of patients with recurrence before 2 years, higher ECOG status, and significantly fewer HPV + patients (all *p* < 0.001). Tumors in the HN-PET-CT cohort had significantly larger volume, a higher proportion of patients treated with chemotherapy and significantly higher 2-year OS (all *p* < 0.001). CT scanner parameters across cohorts are shown in Supplementary Table 3. No statistically significant differences in age, sex, radiation dose, number of fractions, and 2-year FFDM were observed across cohorts.

**Table 1:** Demographic, clinical and dichotomized endpoint data for included patients of the training and test cohorts. Continuous variables are indicated by median [interquartile range]. Categorical variables are indicated with their number and percentage per category. Statistical comparisons between training and each test cohort are also shown. Missing categories/variables within one of the cohorts are indicated by NA. T stage, N stage and Staging were acquired following the American Joint Committee on Cancer 7 th Edition criteria. Number of patients dropped for each 2-year dichotomized prognostic endpoint are labelled as Dropout

		**RADCURE train** **(*n* = 1464)**	**RADCURE** **test** **(*n* = 606)**	***p*-value**	**HN1** **(*n* = 131)**	***p*-value**	**HN-PET-CT** **(*n* = 284)**	***p*-value**
**Age (years)**		63.20 [55.70–71.60]	63.65 [57.20–70.10]	0.76	62.00 [56.00–67.50]	0.10	63.00 [57.00–70.25]	0.86
**Total Dose (Gy)**		70.00 [64.00–70.00]	70.00 [70.00–70.00]	1.00	68.00 [68.00–70.00]	1.00	NA	NA
**Dose fractions** **(# fractions)**		35 [33–35]	35 [35, 35]	1.00	34 [34, 35]	1.00	NA	NA
**GTVp (cm**^**3**^**)**		14.55 [5.00–30.52]	13.68 [4.87–30.14]	0.66	14.15 [3.69–33.71]	0.46	23.72 [11.58–40.55]	** < 0.001**
**Gender**	Male	1200 (82.0)	501 (82.7)	1.00	106 (80.9)	0.86	219 (77.1)	0.067
	Female	264 (18.0)	105 (17.3)		25 (19.1)		65 (22.9)	
**ECOG Status**	ECOG-0	878 (60.0)	335 (55.3)	** < 0.001**	60 (49.6)	** < 0.001**	NA	NA
	ECOG-1	408 (27.9)	248 (40.9)		57 (47.1)		NA	
	ECOG-2	131 (9.0)	20 (3.3)		3 (2.5)		NA	
	ECOG-3	27 (1.8)	3 (0.5)		1 (0.8)		NA	
	ECOG-4	5 (1.0)	0 (0.0)		0 (0.0)		NA	
	Unknown	15 (0.3)	0 (0.0)		0 (0.0)		NA	
**Site**	Oral Cavity	70 (4.8)	10 (1.7)	** < 0.001**	0 (0.0)	** < 0.001**	0 (0.0)	** < 0.001**
	Pharynx	0 (0.0)	0 (0.0)		0 (0.0)		0 (0.0)	
	Hypopharynx	110 (7.5)	28 (4.6)		0 (0.0)		13 (4.6)	
	Larynx	534 (36.5}	172 (28.4)		0 (0.0)		45 (15.8)	
	Nasal Cavity	27 (1.8)	18 (3.0)		46 (35.1)		0 (0.0)	
	Oropharynx	700 (47.8)	362 (59.7)		85 (64.9)		190 (66.9)	
	Other	23 (1.6)	16 (2.6)		0 (0.0)		28 (9.9)	
	Unknown	0 (0.0)	0 (0.0)		0 (0.0)		8 (6.1)	
**T stage**	T1	292 (20.0)	119 (19.6)	0.39	33 (25.2)	** < 0.001**	36 (12.7)	**0.02**
	T2	453 (30.9)	210 (34.7)		32 (24.4)		101 (35.6)	
	T3	425 (29.0)	160 (26.4)		23 (17.6)		92 (32.4)	
	T4	268 (18.3)	107 (17.7)		43 (32.8)		46 (16.2)	
	Unknown	26 (1.8)	10 (1.6)		0 (0.0)		9 (3.2)	
**N stage**	N0	621 (42.4)	203 (33.5)	**0.002**	58 (44.3)	0.25	59 (20.8)	** < 0.001**
	N1	109 (7.5)	55 (9.1)		16 (12.2)		38 (13.4)	
	N2	672 (45.9)	322 (53.1)		54 (41.2)		164 (57.7)	
	N3	62 (4.2)	26 (4.3)		3 (2.3)		19 (6.7)	
	Unknown	0 (0.0)	0 (0.0)		0 (0.0)		0 (0.0)	
**Staging**	I	223 (15.2)	69 (11.4)	**0.005**	24 (18.3)	0.39	NA	NA
	II	194 (13.3)	78 (12.9)		11 (8.4)		NA	
	III	236 (16.1)	75 (12.4)		22 (16.8)		NA	
	IV	810 (55.3)	381 (62.9)		74 (56.4)		NA	
	Unknown	1 (0.0)	3 (0.4)		0 (0.0)		NA	
**HPV16**	Positive	459 (31.4)	290 (47.9)	0.31	22 (16.8)	** < 0.001**	71 (25.0)	0.32
	Negative	232 (15.8)	127 (21.0)		56 (42.7)		45 (15.8)	
	Unknown	773 (52.8)	189 (31.2)		53 (40.5)		168 (59.2)	
**Contrast**	Yes	771 (52.7)	521 (86.0)	** < 0.001**	0 (0.0)	** < 0.001**	0 (0.0)	** < 0.001**
	No	693 (47.3)	85 (14.0)		131 (100.0)		284 (100.0)	
**Chemotherapy**	Yes	976 (66.7)	354 (58.4)	** < 0.001**	34 (26.0)	** < 0.001**	236 (83.0)	** < 0.001**
	No	488 (33.3)	252 (41.6)		97 (74.0)		48 (17.0)	
**2-year LRC**	Recurrent	159 (10.9)	69 (11.4)	0.14	27 (20.6)	**0.003**	29 (10.2)	0.50
	Non-recurrent	1063 (72.6)	361 (59.6)		88 (67.1)		229 (80.6)	
	Dropout	242 (16.5)	176 (29.0)		16 (12.2)		26 (9.2)	
**2-year OS**	Dead	294 (20.1)	89 (14.7)	0.38	30 (22.9)	0.52	26 (9.2)	** < 0.001**
	Alive	1167 (79.7)	401 (66.2)		101 (77.1)		241 (84.9)	
	Dropout	3 (0.2)	116 (19.1)		0 (0.0)		17 (5.9)	
**2-year FFDM**	Metastatic	147 (10.0)	61 (10.1)	0.22	7 (5.3)	0.17	29 (10.2)	0.90
	Non-metastatic	1098 (75.0)	367 (60.6)		96 (73.3)		228 (80.3)	
	Dropout	219 (15.0)	178 (29.3)		28 (21.2)		27 (9.5)	

### Performance of the clinical baseline model

First, we evaluated the predictive performance of the clinical baseline models (Suppl. Tables 4–6). For 2-year OS the model showed AUROCs of 0.83 [0.78–0.88], 0.82 [0.73–0.89] and 0.78 [0.70–0.86] for the RADCURE test, HN1 and HN-PET-CT cohorts. For 2-year LRC, the model showed AUROCs of 0.81 [0.76–0.86], 0.79 [0.69–0.86] and 0.74 [0.66–0.82] for RADCURE test, HN1 and HN-PET-CT respectively. Finally, the model showed AUROCs of 0.76 [0.70–0.82], 0.86 [0.78–0.93] and 0.74 [0.66–0.81] for the RADCURE test, HN1 and HN-PET-CT cohorts for 2-year FFDM.

### Performance of the handcrafted radiomics model

Secondly, we assessed the performance of the handcrafted radiomics models (Suppl. Tables 7–9). For 2-year OS the AUROCs were of 0.73 [0.68–0.80], 0.73 [0.64–0.85] and 0.67 [0.56–0.79] for the RADCURE-test, HN1 and HN-PET-CT cohorts. For 2-year LRC, AUROCs were 0.68 [0.62–0.76] for RADCURE-test, 0.73 [0.60–0.84] for HN1 and 0.63 [0.52–0.74] for HN-PET-CT. For 2-year FFDM, AUROCs were 0.71 [0.64–0.77], 0.68 [0.52–0.83] and 0.67 [0.56–0.79] for RADCURE test, HN1 and HN-PET-CT respectively. Models trained using the subgroups of non-CE (Suppl. Tables 10–12) and CE patients (Suppl. Tables 13–15) did not show significantly higher performance (p $\ge$ 0.20) when compared to the models trained on the entire training cohort (Suppl. Table 16).

### Deep learning models classify 2-year events for OS, LRC and FFDM

**Fig. 3:**
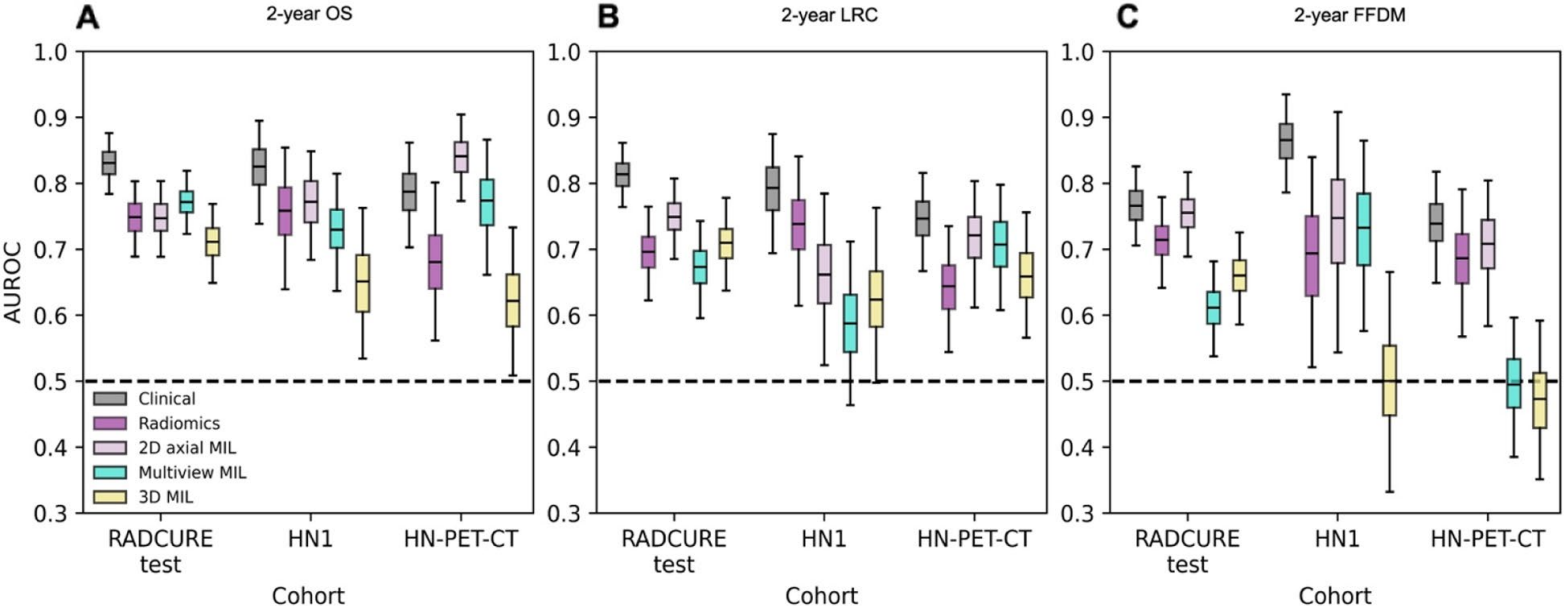
Performance summary for 2-year endpoint classification for the clinical baseline, handcrafted radiomics and the best 2D axial, multiview and 3D MIL models shown across the three test cohorts**.** AUROC distributions from 1000 class-stratified bootstraps are shown for (**A**) 2-year OS, (**B**) 2-year LRC and (**C**) 2-year FFDM

For MIL models, performances for the best models per model type and endpoint are shown in Fig. 3 and Supplementary Table 17 alongside handcrafted radiomics and clinical models. Multiview and 3D models’ performances were not significantly higher than 2D MIL models with axial features (p $\ge$ 0.15, Suppl. Table 18). For MIL models with 2D axial features, the best model for 2-year OS showed AUROCs of 0.75 [0.69–0.80], 0.77 [0.68–0.85] and 0.84 [0.77–0.90] for the RADCURE test, HN1 and HN-PET-CT cohorts respectively. For 2-year LRC, AUROCs of 0.75 [0.68–0.81], 0.66 [0.52–0.78] and 0.72 [0.61–0.80] were obtained for the RADCURE test, HN1 and HN-PET-CT cohorts respectively. Finally, for 2-year FFDM, the best-performing model achieved AUROCs of 0.78 [0.71–0.84], 0.75 [0.55–0.92] and 0.71 [0.59–0.80] for each test cohort. ROC curves across all extractors and endpoints are shown in Suppl. Figure 1–3, showing similar performance for all extractors. Contrast subgroup-specific models did not significantly outperform models trained on the entire training cohort (p $\ge$ 0.20, Suppl. Table 19). When compared against handcrafted radiomics models as shown in Table 2, 2D MIL models showed overall higher but not significantly different (p $\ge$ 0.15) performances except for 2-year OS in the HN-PET-CT cohort where 2D MIL significantly outperformed handcrafted radiomics (p $=$ 0.012).

**Table 2:** AUROC across 2-year endpoints and cohort for the obtained radiomics models alongside *p*-value of one-sided permutation tests with alternative hypothesis of superiority of MIL models over radiomics models

Endpoint	Cohort	AUROC [95% CI] 2D MIL	AUROC [95% CI] radiomics	*p*-value
2-year OS	RADCURE test	0.75 [0.69–0.80]	0.74 [0.68–0.80]	0.50
2-year OS	HN1	0.77 [0.68–0.85]	0.74 [0.64–0.85]	0.50
2-year OS	HN-PET-CT	0.84 [0.77–0.90]	0.68 [0.56–0.79]	**0.012**
2-year LRC	RADCURE test	0.75 [0.68–0.81]	0.69 [0.62–0.76]	0.15
2-year LRC	HN1	0.66 [0.52–0.78]	0.74 [0.61–0.84]	0.84
2-year LRC	HN-PET-CT	0.72 [0.61–0.80]	0.64 [0.53–0.74]	0.15
2-year FFDM	RADCURE test	0.78 [0.71–0.84]	0.71 [0.64–0.77]	0.21
2-year FFDM	HN1	0.75 [0.55–0.92]	0.68 [0.52–0.83]	0.48
2-year FFDM	HN-PET-CT	0.71 [0.59–0.80]	0.67 [0.66–0.79]	0.53

### Deep learning MIL scores stratify patients for time-to-event analysis

**Fig. 4:**
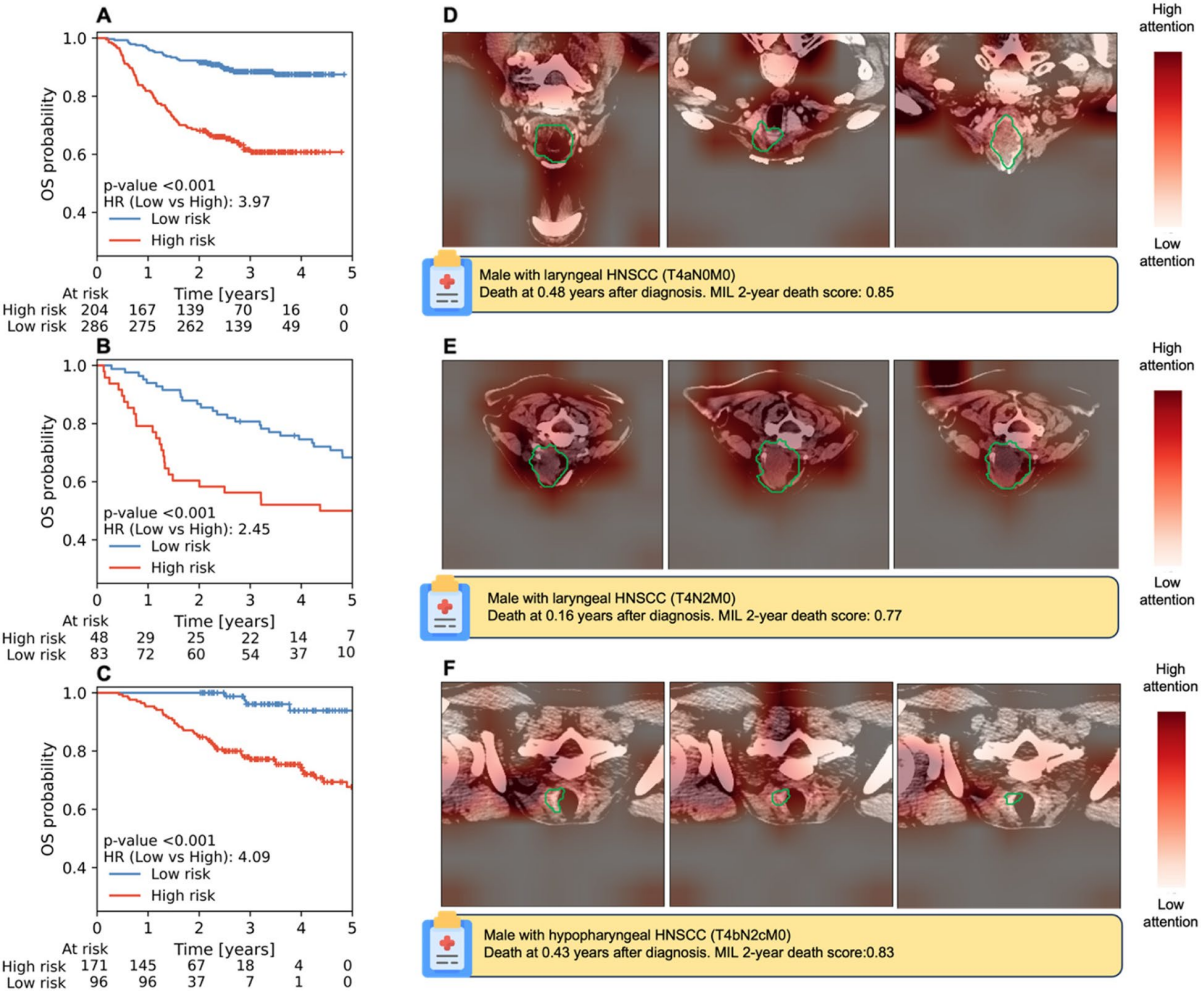
KM plots for OS stratification with the best 2D MIL model for the (**A**) RADCURE test, (**B**) HN1 and (**C**) HN-PET-CT cohorts alongside exemplary axial CT slices with heatmaps for the three CT slices with highest attention belonging to a high-risk patient for RADCURE test (**D**), HN1 (**E**) and HN-PET-CT (**F**) are shown alongside patient characteristics. CT scans are shown after image preprocessing and with a green contour indicating the GTVp segmentation

Next, we investigated whether MIL models could stratify the full time-to-event endpoints. For the best 2D axial MIL models, OS showed significant stratification (*p* < 0.001) across all three test cohorts as shown in Fig. 4. For LRC and FFDM (Suppl. Figure 4), the 2D axial MIL models were able to significantly stratify all (p $\le$ 0.039) and two out of three cohorts (p $\le$ 0.032), respectively, tending to show higher HRs and more significant stratification when compared to handcrafted-radiomics (p $\le$ 0.20) for OS but not higher than clinical baselines as seen in Table 3. Multiview and 3D models did not show better stratification than 2D MIL models (Suppl. Figure 5–6). KM curves for clinical baseline and handcrafted radiomics models are shown in Suppl. Figure 7–8.

**Table 3:** KM stratification summary with HR [95% CI] between high and low-risk groups across all test cohorts for 2D axial MIL, radiomics and clinical models alongside logrank *p*-value and cutoff extracted from the training cohort. NAs indicate that stratification into two groups produced only one group with events and was thus not possible to assess separation. HRs smaller than 1 indicate that patients with higher risk scores have a smaller probability of event

Model	Endpoint	Cutoff	HR [95% CI] RADCURE-test	Logrank *p*-value	HR [95% CI] HN1	Logrank *p*-value	HR [95% CI] HN-PET-CT	Logrank *p*-value
2D MIL axial	OS	0.48169	3.98 [2.63–6.00]	** < 0.001**	2.45 [1.54–3.91]	** < 0.001**	4.09 [1.85–9.04]	** < 0.001**
2D MIL axial	LRC	0.51809	4.77 [2.84–8.02]	** < 0.001**	3.28 [1.52–7.06]	**0.001**	2.14 [1.02–4.48]	**0.039**
2D MIL axial	FFDM	0.46167	4.24 [2.28–7.88]	** < 0.001**	2.11 [0.50–8.86]	0.30	2.51 [1.05–6.00]	**0.032**
Radiomics	OS	0.43367	4.26 [2.79–6.49]	** < 0.001**	2.01 [1.27–3.27]	**0.003**	1.71 [0.73–4.01]	0.20
Radiomics	LRC	0.44668	2.52 [1.57–4.05]	** < 0.001**	3.66 [1.76–7.64]	** < 0.001**	1.99 [1.04–3.80]	**0.033**
Radiomics	FFDM	0.41091	3.43 [1.97–5.67]	** < 0.001**	5.04 [1.02–25.00]	**0.028**	3.13 [0.75–13.00]	0.098
Clinical	OS	0.37451	5.43 [3.42–8.61]	** < 0.001**	5.31 [2.85–9.91]	** < 0.001**	8.98 [3.84–21.08]	** < 0.001**
Clinical	LRC	0.40972	4.98 [3.03–8.18]	** < 0.001**	20.61 [2.80–150.55]	** < 0.001**	3.61 [1.51–8.59]	**0.002**
Clinical	FFDM	0.39670	4.85 [2.55–9.21]	** < 0.001**	NA	NA	5.73 [1.76–18.63]	**0.001**

### Deep learning MIL scores are significant predictors in multivariate analysis

**Fig. 5:**
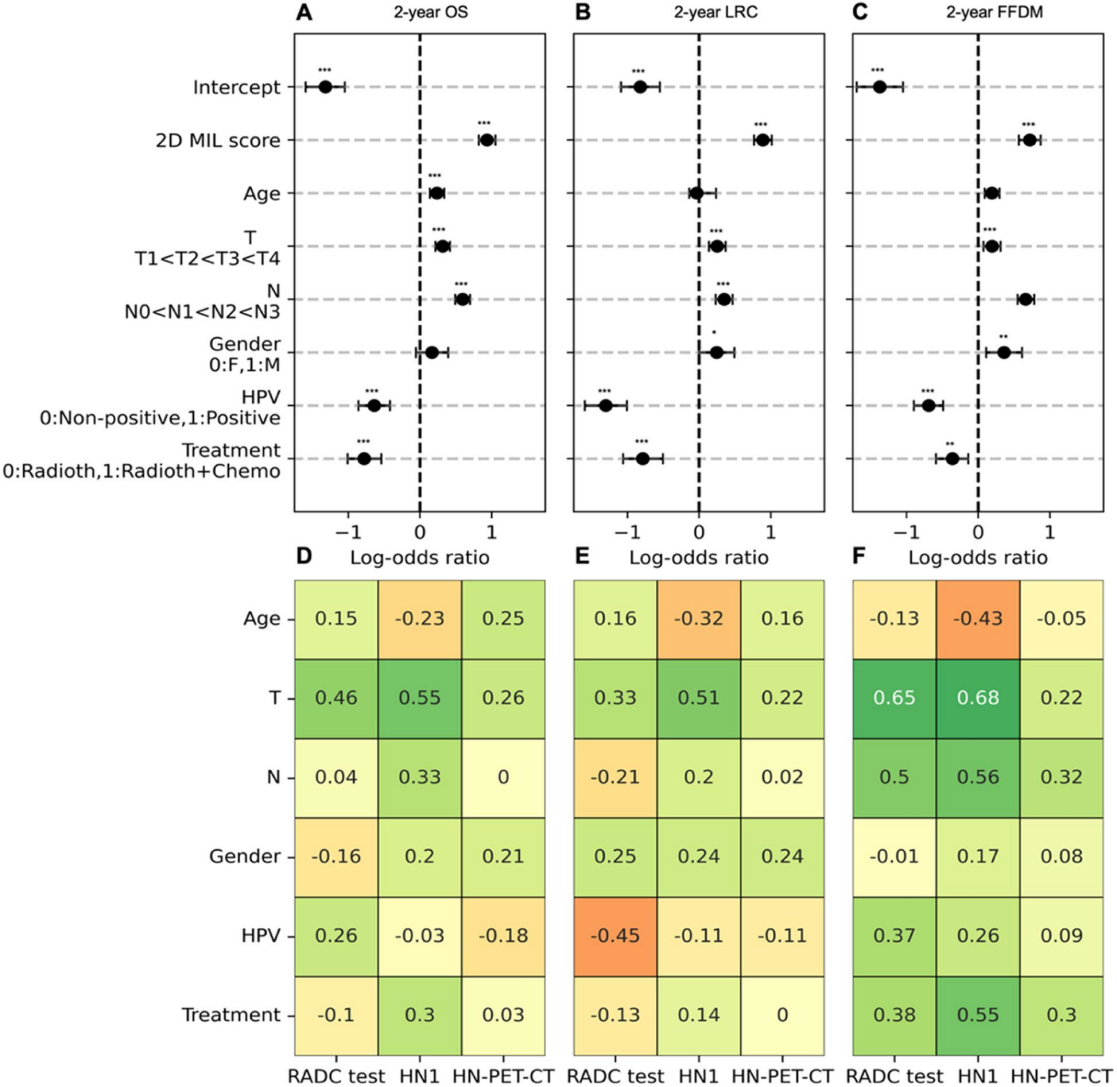
Forest plots for multivariate logistic regression with 2D MIL scores in training (**A**, **B**, **C**) and correlation analysis for test cohorts (**D**,**E**,**F**) per endpoint. Coefficients of multivariate logistic regression (log-odds ratio) are shown for 2-year OS (**A**), 2-year LRC (**B**) and 2-year FFDM (**C**) alongside significance level per coefficient and factoring of variables for analysis. Correlation coefficients of clinical factors through Spearman rho (Age, T stage, N stage) and point-biserial correlation (Treatment, HPV status and gender) are shown for all three test cohorts for 2-year OS (**D**), 2-year LRC (**E**) and 2-year FFDM (**F**) with positive coefficients indicating a positive association between ordering of the clinical factors and increasing 2D MIL scores. Significance levels:.:0.05 < *p* < 0.10, *: 0.01 < *p* < 0.05, **:0.001 < *p* < 0.01, ***: *p* < 0.001

When adjusted for clinical baseline factors in a multivariate setting (Fig. 5A-C) 2D MIL scores remained significant predictors (p $\le$ 0.001), with increases in MIL scores resulting in higher probability of presenting 2-year events for all endpoints (Suppl. Tables 20–22) For 2-year OS, the 2D MIL clinically-adjusted LOR was 0.98 [0.82–1.05], for 2-year LRC LOR was 0.89 [0.77–1.01] and for 2-year FFDM LOR was 0.72 [0.56–0.87]. When examining correlations with clinical factors in the external test cohorts (Fig. 5D-F), MIL scores consistently showed mild to strong correlation with higher T stages for 2-year OS (rho:0.26–0.55), 2-year LRC (rho:0.22–0.51) and 2-year FFDM (rho:0.22–0.65) and consistently showed mild to moderate correlation with N stage for 2-year FFDM (rho:0.32–0.56).

### Multimodal input improves model performance

**Fig. 6:**
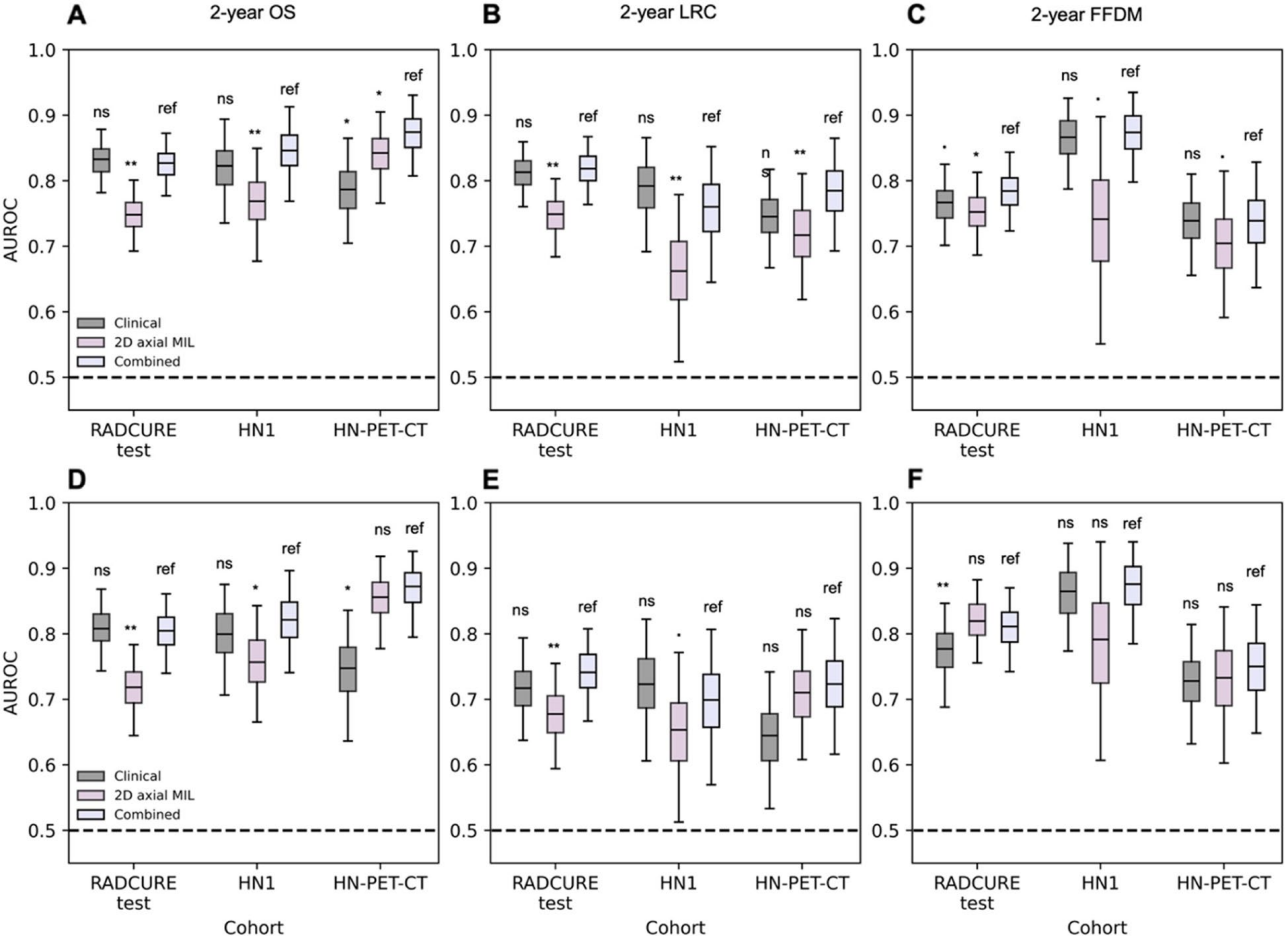
Performance and statistical comparison summary for 2-year endpoint classification for all patients (**A**,**B**,**C**) and patients without HPV-positive HNSCC (**D**,**E**,**F**) in the test cohorts for the clinical baseline, 2D MIL and combined multivariate model with clinical baseline and 2D MIL scores. AUROC distributions from 1000 class-stratified bootstraps are shown across all three test cohorts for (**A**,**D**) 2-year OS, (**B**,**E**) 2-year LRC and (**C**,**F**) 2-year FFDM. Significance levels: ns: non-significant,**.**:0.05 < *p* < 0.10, *: 0.01 < *p* < 0.05, **:0.001 < *p* < 0.01, ***: *p* < 0.001

Finally, we investigated multimodal performance enhancement through combining the clinical baseline and 2D MIL scores. When tested on the external test sets, as shown in Fig. 6 and Supplementary Table 23, while models combining clinical baseline features and MIL scores tended to display similar performances compared to the clinical baseline model across endpoints, significant performance improvements were observed (Fig. 6A-C) for 2-year OS in the HN-PET-CT cohort (0.88 [0.81–0.93] vs 0.78 [0.70–0.86], *p* = 0.018). For the subgroup analysis in non-HPV + patients (Fig. 6D-F), significantly higher performances were observed in the HN-PET-CT cohort for 2-year OS (0.87 [0.79–0.92] vs 0.75 [0.63–0.83], *p* = 0.018) and in the RADCURE test cohort for 2-year FFDM (0.82 [0.74–0.87] vs 0.77 [0.69–0.85], *p* = 0.006).

## Discussion

FMs are an emerging area of research in medical AI and have shown great potential as very flexible feature extractors for a variety of downstream tasks in domains such as histopathology [33]. This study is the first to evaluate the use of FMs as feature extractors within a MIL framework for clinical endpoint prediction in HNSCC using pretreatment CT images. While approaches similar to MIL with DL are not unknown in the radiological domain, such as with brain MRI for Alzheimer's disease prediction with multiple Transformers [34], these studies do not apply their models to cancer prognosis and the models require training for their image feature extractors. In contrast, our study leverages frozen extractors, including pretrained and focuses on downstream tasks. We further benchmarked our models against both handcrafted-radiomics and clinical baselines within a multicentric external validation setting.

We have shown that 2D MIL models achieved good to strong classification performance across all 2-year endpoints for HNSCC patients. Their performance was comparable to the best DL image models in previous RADCURE studies [26] for 2-year OS (0.75 vs. 0.76) and exceeded that of the CNN from Diamant et al. [35] for OS (0.77 vs 0.70) and matched it for LRC (0.66 vs 0.65) on the HN1 cohort. Compared to the CNN from Mateus et al. [36], our 2D MIL models showed higher performance for LRC (0.66 vs 0.45) and OS (0.77 vs. 0.67) on the HN1 test cohort, and outperformed it on OS performance in the HN-PET-CT cohort (0.84 vs 0.82). The models also achieved significant stratification of patients into risk groups, particularly for OS and FFDM, indicating that 2D MIL approaches are competitive for both endpoint prediction and risk-group stratification, showing broader stratification for LRC than other studies employing 2D models for HNSCC [37].

Information from multiple views of the CT did not result in consistent increases in performance or stratification, suggesting limited relevance of coronal and sagittal information when already considering the axial slices for outcome prediction. When considering the 3D FM by Pai et al. [14], performances across all 2-year endpoints were similar to the ImageNet-pretrained 3D extractor and lower than in 2D models. The cause of this might be both a lower quantity of data used [38] for originally training the 3D FM when compared to other FMs like BioMedClip [13] as well as training mostly on general abdominal and chest lesions, suggesting that FMs trained on specific sections and lesions of the body might be suboptimal for prediction within other body regions. Moreover, we also observed that while the BioMedClip FM backbone showed competitive performance, it did not clearly outperform the ImageNet-pretrained models, which could be attributed to the very heterogeneous nature of the PMC-15 M dataset used for training and the paucity of HNSCC CTs in the dataset. Thus, there is a need for larger, more specialized CT datasets to create higher-performing FMs for clinically-relevant tasks, as well as assessment of FMs outside of their original anatomical region to define their scope.

Handcrafted radiomics generally achieved slightly lower performance for 2-year endpoint prediction and stratification compared to 2D MIL models. This performance disparity is in accordance with Gouthamchand et al. [39] where handcrafted radiomics models were shown to systematically offer slightly lower performances over DL models in HNSCC studies. The automatic inclusion of information of not just the GTVp but also of surrounding context from the CT slice through the DL feature representations might provide information related to aspects such as tumor spread across the slice and explain the observed gains in both classification performance and stratification. While previous studies have included handcrafted radiomics information outside the GTVp [40], showcasing increased performance compared to GTVp-only models, these studies require tuning the size of peritumoral volume to be included, in contrast to DL approaches which can achieve this implicitly.

MIL scores were statistically significant in multivariate analysis and correlated across endpoints with tumor T stage for all external cohorts; indicating that models learned patterns associated with known clinical variables. Correlations of scores to clinical variables were shown to be endpoint-dependent, as positive correlations with N-stage for 2-year FFDM were observed across all three test cohorts which is a known risk factor for distant metastasis in HNSCC [41], while showing little to no correlations to N-stage for 2-year OS MIL scores. Increases in performance for the multimodal models when compared to the clinical baseline were observed both in all patients and in the subset of patients of non-HPV + tumors, indicating potential for MIL approaches for multimodal modeling with clinical baselines. Such increases were, however, not consistent across all endpoints and test cohorts due to collinearity with the clinical baseline. Future directions to reduce overlap and collinearity for improved harmonization of MIL scores with clinical baselines could include bias mitigation techniques [42] or the usage of graph-based neural networks, which Bae et al. [43] have shown to produce models with consistent enhancement of clinical baselines in HNSCC and may be combined with MIL.

The present study is not without its limitations; considered data is from publicly-available retrospective cohorts and further validation is required to extend the modeling scope beyond a proof of concept for the applicability of MIL and FM approaches in HNSCC with pretreatment CTs. Secondly, the paper does not consider handcrafted radiomics features within the peritumoral area. Thirdly, while other 3D FMs based on CT exist such as MERLIN [44] or CT-CLIP [45], they are not evaluated due to non-suitability for MIL modeling. And lastly, the study does not explore applicability of FMs as extractors in settings with low training data for endpoint prediction models or finetuning of FMs for downstream prediction.

In conclusion, the study has shown for the first time the potential of 2D MIL models with FM backbones for competitive endpoint prediction and stratification in HNSCC patients, matching or outperforming handcrafted radiomics models as well as showcasing potential for multimodal enhancement in non-HPV + HNSCC tumors.

## Supplementary Information

Supplementary Material 1.

## Data Availability

All data from the three cohorts described in the present study are available in the TCIA database (https://www.cancerimagingarchive.net/) through submission of a TCIA No Commercial Limited Access License and were used with their corresponding approvals. The code containing the models and feature extraction pipeline are available at: https://github.com/KatherLab/HNSCC_FM_MIL_study. The STAMP code used for the analysis within this paper can be found at https://github.com/KatherLab/STAMP.

## Abbreviations

FM: Foundation model
MIL: Multiple instance learning
HNSCC: Head and neck squamous cell carcinoma
GTVp: Primary gross tumor volume
AUROC: Area under the receiver operator curve
KM: Kaplan.Meier estimator
LOR: Log-odds ratio
HR: Hazard ratio
OS: Overall survival
LRC: Locoregional control
FFDM: Freedom from distant metastasis
HPV +: Human papillomavirus positive
